# Effectiveness of skin interface pressure and temperature monitoring for pressure injury prevention in paraplegic patients: a comparative study

**DOI:** 10.3389/fpubh.2025.1521948

**Published:** 2025-10-07

**Authors:** Aimei Miao, Cai Lin, Yali Ni, Shaobo Yin, Chang Lv, Haohao Huang, Xiaoqiong Jiang, Huifen Zhou

**Affiliations:** ^1^Burn and Wound Healing Centre, The First Affiliated Hospital of Wenzhou Medical University, Wenzhou, Zhejiang, China; ^2^Department of Burn Wound Repair, The First Affiliated Hospital of Wenzhou Medical University, Wenzhou, Zhejiang, China; ^3^Medical Engineering Department, Xinyang 154 Hospital, Xinyang, Henan, China; ^4^Department of Rehabilitation, The Second Affiliated Hospital of Wenzhou Medical University, Wenzhou, China; ^5^School of Nursing, Wenzhou Medical University, Wenzhou, China; ^6^Center Sterile Supply Department, The First Affiliated Hospital of Wenzhou Medical University, Wenzhou, Zhejiang, China

**Keywords:** paraplegia, pressure injury, postural management, skin interface pressure, skin temperature, pressure ulcer prevention

## Abstract

**Purpose:**

Studies indicate that the conventional practice of turning patients every 2 h has certain limitations in preventing pressure injuries among paraplegic patients. This study aimed to explore the application effect of skin interface pressure combined with skin temperature monitoring to prevent pressure injury (PI) in paraplegic patients.

**Methods:**

Using convenience sampling, 89 paraplegic patients from the Department of Rehabilitation, Department of Orthopedics, Wound Center, and Department of Neurology of two tertiary hospitals in Wenzhou (from January 2021 to July 2023) were randomly assigned to the study and control groups. The study group received infrared thermography and portable pressure/temperature monitoring at pressure points, guiding personalized position management plans. In contrast, the control group received routine disease treatment and standard pressure injury protection. Follow-up assessments were conducted at 3-, 6-, and 12-month post-intervention for both groups. Data collected included pressure injury incidence rates at these three time points, patient satisfaction scores, nursing time spent on positioning management during hospitalization, and mean daily frequency of position changes.

**Results:**

The incidence of pressure injury in the study group and the control group at 3, 6, and 12 months after the end of the trial was 2.3% vs. 6.7, 4.5% vs. 13.3, and 4.5% vs. 20%, respectively. Spearman’s correlation analysis found that the sacrococcygeal pressure values of paraplegic patients were positively correlated with the occurrence of pressure injuries (*r* = 0.676), while the skin temperature values were negatively correlated with pressure injuries (*r* = −0.701). The degrees of patients’ satisfaction with nursing in the study group were significantly higher than those of the control group. Furthermore, the nursing workload of the study group was considerably less than that of the control group.

**Conclusion:**

This study implied that personalized positioning guided by real-time sacral pressure and skin temperature monitoring significantly reduced long-term PI incidence in paraplegic patients. Pressure and temperature may serve as validated early-warning indicators. Our findings supported replacing rigid 2-h schedules with individualized protocols.

## 1 Introduction

Pressure injury (PI, also named pressure ulcer before 2016) is defined as a localized injury on the skin and/or subcutaneous soft tissues due to prolonged pressure or shear, or both (1, 2). PI usually occurs at bony prominences, areas of contact with medical devices or other equipment (3). Paraplegic patients are prone to PI due to the loss of sensory-motor movement below the injury area, the absence of a protective response at the site of skin compression, cutaneous vasculo-neurological dysfunction, as well as localized moisture and contamination of the skin (4, 5). A previous study showed that the prevalence of PI was 49.2% in paraplegic patients (6). Felder JM et al. (7) reported that 23.7 and 39.5% of paraplegic and quadriplegic patients undergoing rehabilitation in the United States had PI in at least one site, and 85.7% of paraplegic patients in Japan had PIs, and 17.9% had refractory PIs. Timed position management as a simple and effective means to reduce PI is widely used in clinical care (8). However, the conventional 2-h turning (i.e., position management) has several limitations in preventing PIs in paraplegic patients, that is, difficulties in clinical practice, uneven distribution of stress, neglect of individual differences, and impact on patients’ quality of life (9). The sacrum, ischial tuberosities, and trochanteric regions are the most commonly affected sites due to prolonged pressure and immobility. The high local prevalence is influenced by factors such as limited access to specialized wound care, insufficient caregiver training, and inconsistent follow-up after discharge. Current practices to prevent PI include regular repositioning schedules, use of pressure-relieving surfaces, skin inspection protocols, nutritional support, and patient and caregiver education. Despite these practices, several challenges hinder effective PI prevention, including limited resources, low patient adherence, and inconsistent training and protocol enforcement. Routine treatment for pressure injuries involves stage-specific wound care—including cleansing, appropriate dressings, debridement, and pressure relief—along with nutritional support, infection control, and patient education to promote healing and prevent recurrence. Positional management has been used to improve the prevention of PIs by monitoring pressure at the pressure site (10). In addition, studies have shown that infrared thermography can accurately monitor abnormal changes in skin temperature in the early stages of pressure injury, thereby alerting the clinic to take early preventive measures (11).

Countries such as the United States and Sweden have demonstrated the efficacy of pressure monitoring at patient interfaces to guide positioning management in preventing pressure injuries. Furthermore, studies indicate that infrared thermography enables the precise detection of abnormal skin temperature changes during early stage pressure injuries, prompting timely clinical interventions. Based on these findings, this study hypothesizes that integrating pressure monitoring with skin temperature assessment at pressure interfaces in paraplegic patients may enable earlier detection of subclinical signs, thereby optimizing positioning protocols and enhancing pressure injury prevention efficacy. During follow-up, data collected included pressure injury incidence rates at these three time points, patient satisfaction scores, nursing time spent on positioning management during hospitalization, and mean daily frequency of position changes.

## 2 Methods

### 2.1 Study population

Prior to commencement, this study was approved by the Medical Ethics Committee. Written informed consent was obtained from all enrolled patients or their designated legal guardians/relatives. A convenience sampling method was used to select paraplegic patients from January 2021 to July 2023 in the Department of Rehabilitation, Department of Orthopedics Department, Wound Center, and Department of Neurology of The First Affiliated Hospital of Wenzhou Medical University in Wenzhou City, China. The inclusion criteria are listed as follows: ① Patients who met the diagnostic criteria for paraplegia and whose injury plane was below the thoracic segment; ② Age 18–65 years old; ③ Estimated length of hospital stay ≥20 days; ④ After the researcher explained the purpose of the study, the patients, as well as their family members (immediate family members or guardians), and caregivers were willing to participate in the study. The exclusion criteria are listed as follows: ① Patients, family members or caregivers with psychiatric diseases, severe sepsis, diabetes, hypertension, uremia, etc., diagnosed with multiple organ failure, advanced cancer, and terminal patients, patients, family members, or caregivers who are unable to communicate verbally; ② Patients who have already suffered from pressure injuries or have pre-symptomatic symptoms of pressure injuries prior to entering the study; ③ Those who are suffering from diseases affecting skin observation; ④ Those who are medically instructed to braked or prohibited from turning over; ⑤ Those who are undergoing surgical treatment during hospitalization. Random number table method is adopted to divide the participants into the control group (routine care) and the study group (pressure interface pressure combined with skin temperature monitoring). To ensure ethical compliance and research transparency, this study obtained formal approval from the Medical Ethics Committee (ethical approval number: KY2024-065). Furthermore, written informed consent was secured from all participating patients or their legally authorized representatives (immediate relatives or guardians) prior to inclusion, upholding the principles of participant autonomy and research validity.

### 2.2 Intervention

#### 2.2.1 Control group

During hospitalization, patients received standard disease treatment and pressure injury prevention care. Within 1–2 days prior to discharge, specialized nurses assessed all patients’ activities of daily living (ADL) and Braden Scale risk scores, provided guidance on conventional 2-h repositioning schedules, and delivered primary pressure injury care training to patients and primary caregivers. This training covered positioning techniques based on natural care principles (including turning and maintenance), identification of high-risk anatomical sites and vulnerable time periods, risk factors and prevention strategies, nutritional support, proper skin assessment, and early recognition/management of skin changes. Post-discharge, all participants received home-visit follow-ups with reinforcement of positioning management and prevention guidance at 3, 6, and 12 months.

#### 2.2.2 Study group

The study group implemented a new nursing strategy that combines pressure at the pressure interface with skin temperature monitoring and skin assessment, serving as a comprehensive early warning to guide personalized positional management. This approach encompasses several key aspects discussed in the following.

1. Pressure interface pressure combined with skin temperature monitoring

The patients lay on the ordinary medical jet air mattress bed during the study period. All the measurement sessions should avoid the operations that affect the measurement results. The following actions are prohibited for 1 h before the measurement, that is, physical therapy, transcutaneous electrical stimulation of neural therapy, ultrasound therapy, acupuncture, physical stimulation, hot or cold compresses. Activities, eating, washing, bathing, cleaning, and disinfecting of the measurement site are prohibited for 30 min before the measurement. During the measurement process, keep the imaging lens of the infrared thermal camera, the pressure sensor probe, and the sacrococcygeal area in the same position, with the focus at the bony protuberance of the sacrococcygeal region. The following principles were followed: fixed instrument, fixed time, fixed distance, and fixed observer. The average value was taken as the final result.

**Figure 1:**
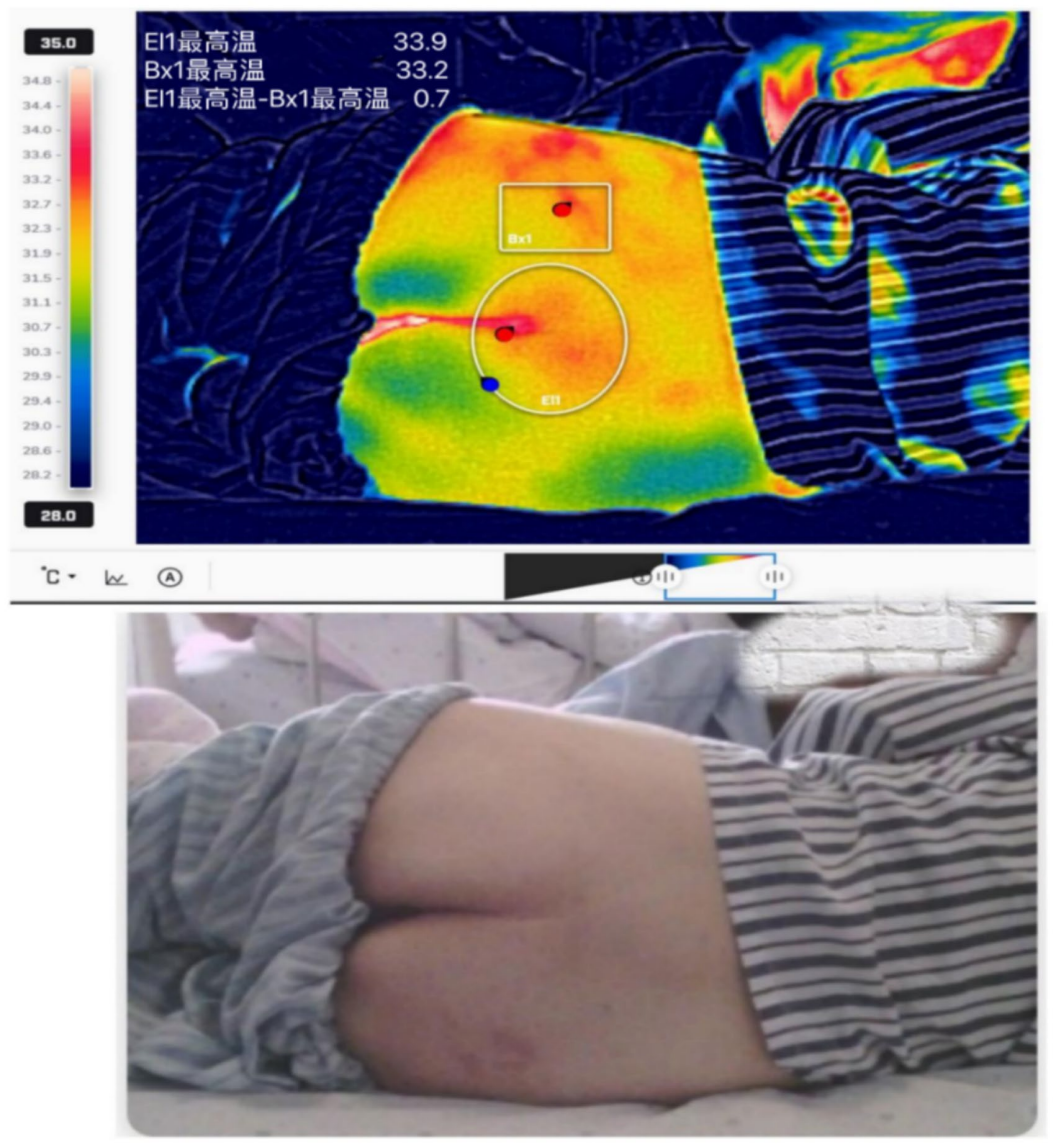
Thermogram showing uniformly distributed skin temperature within the normal range at the pressure-bearing site.

**Figure 2:**
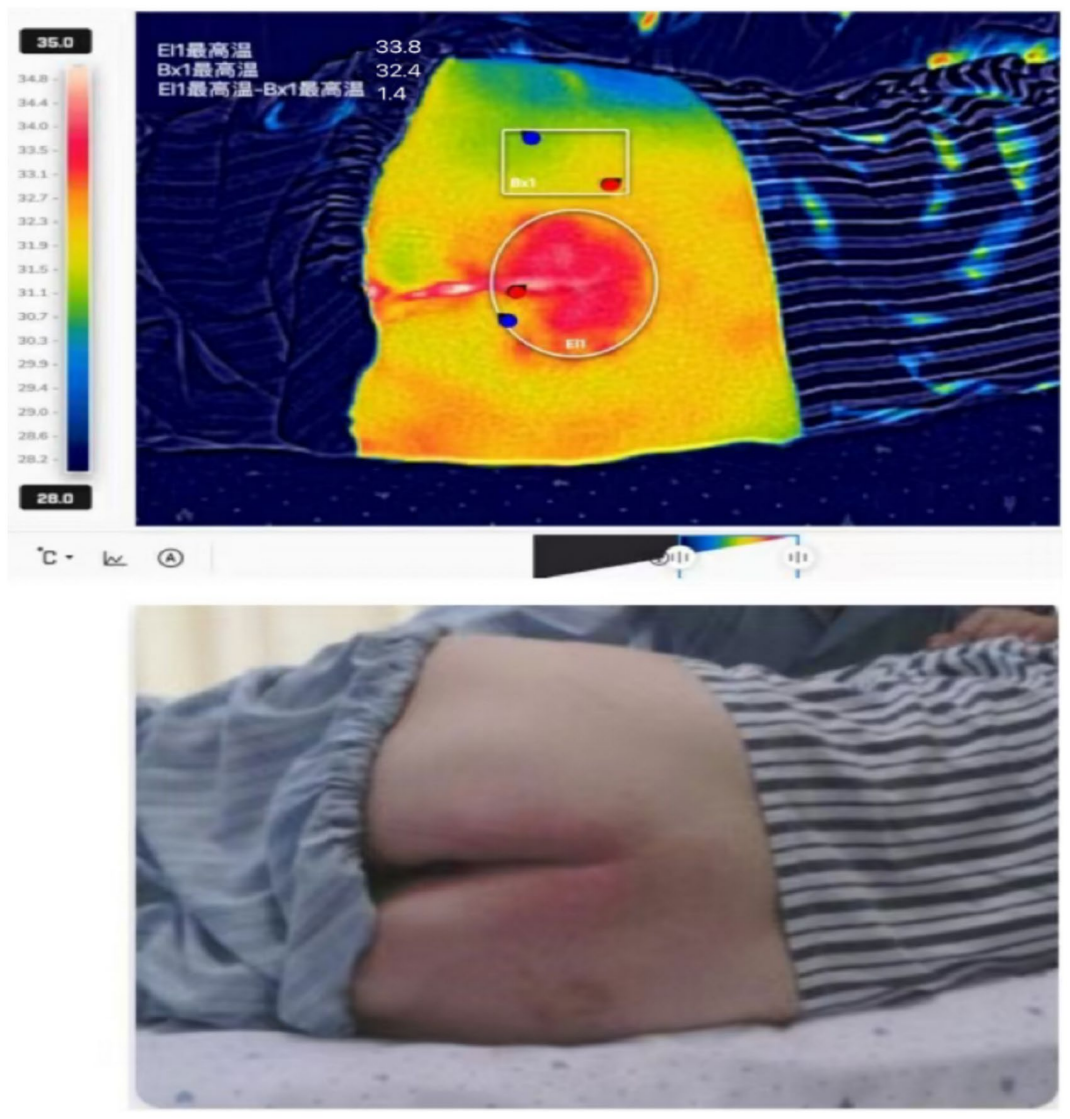
Thermogram showing abnormally elevated skin temperature at the pressure-bearing site.

1. Design an individualized position change program for different paraplegic patients

- Pilot study to determine early warning thresholds for pressure and skin temperature differential in the sacrococcygeal region: Forty-one paraplegic patients meeting the inclusion/exclusion criteria (Section 1.1) were enrolled in this pilot study after providing informed consent. Sacrococcygeal interface pressure and skin temperature differential were measured daily for 10 days using a portable pressure monitor and infrared thermography (methodology consistent with Section 1.2.2.1). Pressure injuries (PIs) were assessed by two specialist nurses using standard criteria. Receiver operating characteristic (ROC) curve analysis was employed to determine early warning thresholds for pressure and skin temperature differential to guide postural management. Five patients (12.1%) developed pressure injuries during the pre-intervention phase. Following the implementation of shortened repositioning intervals, all patients showed improvement and were successfully discharged. ROC curve analysis established the following early warning thresholds: (1) Pressure: 63 mm Hg (sensitivity, 0.800; specificity, 0.861; and maximum Youden’s index 0.661). (2) Skin temperature differential: 1.40 °C (sensitivity, 0.800; specificity, 0.972; and maximum Youden’s index 0.772). Clinical interpretation of thermography: (1) Normal circulation: Thermographic images showing uniform skin temperature distribution within the normal range and a relative temperature differential <1.4 °C (Figure 1) indicate adequate skin blood perfusion under the current position, suggesting the repositioning interval may be safely extended. (2) Impaired circulation/Risk: Thermographic images showing abnormally elevated localized skin temperature and a relative temperature differential ≥1.4 °C (Figure 2) suggest potential tissue compression and impaired blood flow. Immediate repositioning is required to prevent skin damage.
- Repositioning protocol during hospitalization: The in-hospital repositioning protocol underwent a progressive schedule as follows: Days 1–10, 11–13, 14–16, and 17–20 were conducted by repositioning every 2, 2.5, 3, and 3.5 h, respectively. Contingency Protocol: If any of the following occurred during the schedule, including mild erythema at pressure sites, pre-injury symptoms, pressure/skin temperature exceeding thresholds (63 mm Hg or 1.40 °C), the repositioning interval was immediately reduced to the previous phase’s duration (e.g., from 3.5 to 3.0 h). After maintaining this adjusted interval for 3 days with continued monitoring of skin status and pressure injury development, the progressive schedule was discontinued. Standard repositioning continued at the modified interval. Should warning signs reappear, the interval was again reduced to the prior phase’s duration.
- Home-based repositioning protocol: Patients maintained the final hospital phase schedule (3.5-h repositioning intervals). Trained family members (hospital-certified through consent training) measured sacrococcygeal pressure and skin temperature daily using hospital-provided instruments. Threshold-driven adjustments: Shorten the interval by 0.5 h if measurements exceed thresholds; lengthen the interval by 0.5 h when measurements remain below thresholds for ≥24 h. Emergency response: Any observed non-blanchable erythema, persistent pressure marks, or suspected tissue compromise requires photographic documentation of the affected area, immediate telehealth consultation via the hospital’s “Internet + Nursing” platform, and specialist-guided intervention (video instruction, clinic visit, or home nursing deployment). Structured follow-up for months 1, 2–3, 4–6, and 7–12 was weekly, biweekly, monthly, and quarterly home visits. The visit objectives included skin integrity assessment, technique verification (temperature/pressure measurement), equipment calibration, and on-demand nursing support.
- In case of skin abnormalities during the testing period, the interval time will be dynamically adjusted

It may terminate the next longer interval time test when skin abnormalities persist for 2 days, occur more than 2 times a day, or non-human or deterioration of the condition caused by more than 1 stage of pressure damage manifestations such as skin redness, pressure, skin temperature, and other significant changes. If there is no effective improvement or the skin damage progresses or is aggravated, the treatments may be withdrawn.

1. Personalized positioning management in three positions (i.e., semi-sitting, lateral, and supine)

Patients were changed every 2 h on days 1–7, alternating between lateral, supine, and semi-sitting positions, and were closely observed. If the patient does not exhibit to have mild redness of the pressured skin (which can be faded within 30 min after decompression) for 2 days of observation, the turning interval will be extended by another 30 min until the position change is necessary (e.g., defecation and treatment). After the position change, the patient is assisted with appropriate decompression devices to carry out effective pressure dispersal care, so as to keep the patient in a well-positioned position, and dynamic fine-tuning will be carried out when necessary.

### 2.3 Investigative tools

#### 2.3.1 Japan Palm Q portable pressure tester

Application of the Japan Palm Q portable pressure tester to monitor the corresponding point pressure. The measurement area sensing diameter was 10 × 10 cm with five sensing points. The material was soft and easily fit into the patient’s body, featuring a curved surface designed for monitoring bone protrusion. The measurement method for identifying peak pressure points across different lying positions: Nursing staff will position the sensor pad over the area anticipated to exhibit the highest pressure, adjusting it as needed to center the pad over the point of peak pressure. The pad will remain in place for 2 min, ensuring that boundary effects do not compromise the pressure readings. Pressing the start button to start measuring, after about 12 s, the sensor pad’s number and chart will flash, showing the measured pressure value of the interface.

#### 2.3.2 FLIR ONE pro infrared thermal imaging camera external probe (USA)

The FLIR ONE Pro Infrared thermal imaging camera is used to obtain the temperature of the patient’s corresponding monitoring point. The device has an optical camera and an infrared camera, each connected to the mobile device. The FLIR One supporting software enables shooting in various modes, including visible light images, ordinary thermal images, and dynamic thermal image enhancement. The temperature measurement ranges from −20 to 400 °C. The temperature resolution is 0.1 °C. Up to 3 movable temperature measurement points and 6 movable temperature measurement zones are displayed on the screen simultaneously. Before measurement, the observation area was exposed for 4 min to exclude local reversible ischemia and reactive congestion caused by pressure. At the same time, it can dissipate the heat generated by the contact between the skin surface and the mattress during the period of lying on the bed. The infrared thermal image of the pressure interface was taken, and the temperature data were read by FLIR Tools software package.

#### 2.3.3 General information questionnaire

A general information questionnaire for paraplegic patients was designed to collect general information about the patients, including sex, age, marital status, education level, cause of paraplegia, spinal cord injury segment, history of paraplegia, and degree of spinal cord injury (ASIA classification). The comorbid chronic diseases were evaluated by the Charlson Comorbidity Index (CCI), aiming to assess the severity of the patients’ disease.

#### 2.3.4 Homemade record cards for positional management of paraplegic patients

Homemade position management record card for paraplegic patients featuring entries that include date, time, position (lateral, semi-sitting, and supine), pressure and temperature at corresponding monitoring points, skin condition, interval between position changes, previous position, caregiver’s signature, and remarks column.

#### 2.3.5 Braden pressure injury rating scale

The Braden Pressure Injury Rating Scale evaluates six aspects of sensation, activity patterns, moisture, nutrition, mobility, friction, and shear. Each of these scores ranges from 1 to 4, with “friction and shear” scored from 1 to 3. The lower the score, the higher the risk of pressure ulcers.

#### 2.3.6 Nutritional risk screening 2002 (NRS-2002)

The NRS-2002 was used to assess the nutritional status of paraplegic patients. The scale was developed by experts from the Danish Society for Parenteral Enteral Nutrition. It includes a nutritional status score (0–3 points), a disease severity score (0–3 points), and an age score (plus 1 point for those 70 years of age and older).

#### 2.3.7 Satisfaction and nursing workload

The assessment of patient satisfaction was designed according to a study in China (12). This questionnaire assesses nursing satisfaction by categorizing responses into three groups: very satisfied, satisfied, and dissatisfied, with a total of three scores (satisfaction rate = [very satisfied + satisfied]/total cases × 100.00%). The Nursing Workload Statistics Form primarily focused on the time required for positional management performed by nursing staff or caregivers for patients during hospitalization, as well as the labor intensity, specifically measured by the average number of position changes per patient per day (13).

### 2.4 Data collection methods

The patient’s general condition form, the paraplegic patient’s position management record form (including monitoring time point, lying position, monitoring site, pressure, skin temperature, and skin condition) were recorded and collected. The staging of pressure injuries was diagnosed according to the 2019 edition of Pressure Injury Prevention and Treatment, which was developed by NPUAP (National Pressure Injury Advisory Panel) and EPUAP (European Pressure Ulcer Advisory Panel) in conjunction with Pan Pacific Pressure Injury Alliance (PPPIA).

In the course of the trial, data on sacrococcygeal pressure and skin temperature were collected and collated from the two groups of patients to compare the incidence of pressure injuries in the two groups, patient satisfaction (by evaluating the subjective feelings of patients brought by the care of the different modes of positional management, such as comfort, pain, and quality of sleep), and the difference in nursing workload (mainly by comparing the time spent by nursing staff or caregivers for patients to do positional management, labor intensity, and the number of times patients changed positions a day). Patients in both groups were followed up at 6 months and 1 year after the trial to collect data on the incidence of PIs.

### 2.5 Quality control

Prior to carrying out the study, adequate and uniform training was given to the researchers to ensure that they understood and were able to carry out the study procedures accurately. The standardized and reliable equipment was used for the tests. The equipment was inspected and maintained regularly to ensure that it operated correctly, thereby ensuring the reliability of the information collected. After coding and checking the data and information, another entrant performs double entry and secondary checking to ensure the accuracy of the data entered.

### 2.6 Statistical analysis

The data were statistically analyzed using Statistical Package for the Social Sciences (SPSS) version 25.0 software. Measurement information obeying a normal distribution was described by mean ± standard deviation. Samples were compared using a two-sample *t*-test. The data that did not obey a normal distribution were described by median and quartile, and rank–sum test was used for comparison between groups. Count data were expressed as cases, percentages, or per cent. Comparisons between groups were conducted using the chi-square test, Fisher’s exact test, or rank–sum test. The test level was set as *α* = 0.05, while *p* < 0.05 was considered statistically significant.

## 3 Results

### 3.1 General information on the study population

There were no statistically significant differences between the two groups of paraplegic patients in terms of sex, age, marital status, education level, cause of paraplegia, spinal cord injury segment, history of paraplegia, degree of spinal cord injury (ASIA classification), Charlson’s disease score, activities of daily living (ADL) score, nutritional status score, and Braden’s score (all *p* > 0.05). These results indicated that the two groups of patients were comparable (Table 1).

**Table 1:** Comparison of baseline data between the two groups of patients (*n* = 89).

Variable	Control group (*n* = 45)	Study group (*n* = 44)	Test statistic	*p*-value
Age (years,^−^χ ± s)	53.62 ± 12.25	57.30 ± 13.56	−1.342	0.183
Sex (*n*, %)			0.131	0.717
Men	28 (62.2)	29 (65.9)		
Women	17 (37.8)	15 (34.1)		
Marital status			2.894	0.089
Married	35 (77.8)	40 (90.9)		
Unmarried/divorced/widowed	10 (22.2)	4 (9.1)		
Education			0.223	0.894
Junior high school and below	35 (77.8)	34 (77.3)		
High school or secondary school	7 (15.6)	6 (13.6)		
College and above	3 (6.7)	4 (9.1)		
Causes of spinal cord injury			0.016	0.900
Contraindication	19 (42.2)	18 (40.9)		
Non-traumatic	26 (57.7)	26 (59.1)		
Spinal cord injury segment			0.169	0.919
Thoracic segment	17 (37.8)	17 (38.6)		
Waist section	15 (33.3)	13 (29.5)		
Sacrococcygeal section (anatomy)	13 (28.9)	14 (31.8)		
Asia impairment classification			0.117	0.990
Grade a	7 (15.6)	7 (15.9)		
Level b	15 (33.3)	14 (31.8)		
Level c	14 (31.1)	15 (34.1)		
Level d	9 (2.0)	8 (18.2)		
Paralysis history			0.729	0.948
Within 3 months	11 (24.4)	11 (25.0)		
3–6 months	4 (8.8)	4 (9.1)		
7–12 months	4 (8.8)	5 (11.4)		
1–2 years	9 (2.0)	6 (13.6)		
More than 2 years	17 (37.8)	18 (40.9)		
Nutritional status score			0.002	0.967
1–3 points	39 (86.7)	38 (86.4)		
4–7 points	6 (13.3)	6 (13.6)		
Ability to perform activities of daily living (ADLS)			0.387	0.824
≤40 points	32 (71.1)	30 (68.2)		
41–60 points	12 (26.7)	12 (27.3)		
61–99 points	1 (2.2)	2 (4.5)		
Braden score [points, M (P_25_, P_75_)]	12 (12, 12)	12 (12, 12)	−0.521	0.473
Charlson Comorbidity Index [points, M (P_25_, P_75_)]	4 (4, 5)	5 (4, 5.75)	−1.162	0.245

### 3.2 Comparison of the incidence of pressure injuries and skin abnormalities in the two groups

Three months after the end of the trial, the incidence of pressure injuries and skin abnormalities in the test group was lower than that in the control group, but without statistical significance (*p* = 0.616, Table 2). However, as shown in Table 3, patients in the study group had a significantly lower rate of pressure injuries and skin abnormalities than those in the control group at 6 months and 1 year after the trial (*p* < 0.001 and *p* = 0.027). In addition, Spearman’s correlation analysis found that the sacrococcygeal pressure values of paraplegic patients were positively correlated with the occurrence of pressure injuries, while the skin temperature values were negatively correlated with pressure injuries. The correlation analysis between pressure and skin temperature and pressure injury is shown in Table 4.

**Table 2:** Comparison of the incidence of skin abnormalities and pressure injuries between the two groups of patients at the end of the trial for 3 months (cases, %).

Groups	Number of examples	Pressure injury	
		Unprecedented	Incidence
Control group	45	42 (93.3)	3 (6.7)
Study group	44	43 (97.7)	1 (2.3)
*χ* ^2^		/	
*p*		0.616	

Fisher’s precision test.

**Table 3:** Comparison of the incidence of pressure injuries in the two groups of patients at 6 months and 1 year at the end of the trial (cases, %).

Groups	*N*	6 months		12 months	
		Unprecedented	Incidence	Unprecedented	Incidence
Control group	45	38 (84.4)	7 (15.6)	36 (80.0)	9 (20.0)
Study group	44	42 (95.5)	2 (4.5)	42 (95.5)	2 (4.5)
*χ* ^2^		54.212		4.905	
*p*		<0.001		0.027	

Fisher’s precision test.

**Table 4:** Analysis of the relationship between pressure and skin temperature and pressure injury in both groups of patients.

	Control group	Study group	*r*	*p*-value
Pressure (mm Hg)	98.10 ± 15.49	90.08 ± 9.15	0.676	0.001
Skin temperature (°C)	38.20 ± 4.53	30.34 ± 5.6	−0.701	<0.001

### 3.3 Comparison of satisfaction

As listed in Table 5, the patient satisfaction profile of the study group was significantly better than that of the control group (*p* = 0.032).

**Table 5:** Comparison of patient’s satisfaction on nursing between the two groups (cases, %).

Groups	*N*	Mostly satisfactory	Satisfaction	Dissatisfied
Control group	45	33 (73.3)	9 (20.0)	3 (6.7)
Study group	44	41 (93.2)	3 (6.8)	0 (0.0)
*χ* ^2^		6.854		
*p*		0.032		

### 3.4 Comparison of nursing workload

Comparison of the nursing workload of the two groups (routine 2-h position change group and personalized position management group) was done mainly by comparing the time needed to be spent by the nursing staff or carers to perform position management for the patients in a day and the number of position changes made by the patients in a day. The results showed that the nursing workload of the study group was significantly less than that of the control group (all *p* < 0.001, Table 6).

**Table 6:** Comparison of nursing workload between the two groups.

Groups	Number of examples	Asana management takes time	Number of times a day the patient changes position
Control group	45	90 (85, 92.5)	12 (11, 12)
Study group	44	55 (51.25, 65)	9 (8.25, 10.75)
*Z*		−7.531	−5.953
*p*		<0.001	<0.001

The patients in the control group were routinely protected by a fixed 2-h position change after the administration of conventional treatment, while the patients in the study group had an individualized position management plan based on the results of pressure and skin temperature monitoring. The results showed that the time interval for patients in the study group to perform position changes was increased, thus reducing the workload of the nursing staff (Table 7).

**Table 7:** Statistics on the number of 44 patients in the test group with different frequencies of position change.

Frequency of position changes	Number of patients
Within 2 h	4
2.1–2.5 h	14
2.6–3.0 h	13
3.1–3.5 h	5
Frequency of dynamic postural changes with relative immobility	8

## 4 Discussion

The present study establishes that continuous monitoring of sacrococcygeal interface pressure and skin temperature to guide personalized repositioning significantly reduces 12-month pressure injury incidence in paraplegic patients, while showing protective trends at earlier timepoints. The strong correlations between elevated pressure/increased skin temperature and subsequent injury development provide physiological validation for using these parameters as real-time biomarkers. Crucially, this evidence supports transitioning from rigid 2-h turning schedules to individualized protocols triggered by objective tissue tolerance thresholds. The significant reduction in nursing workload and improved patient satisfaction further underscore the clinical utility of this approach.

### 4.1 The theoretical frameworks to contextualize our findings

Self-Determination Theory (SDT) posits that autonomy, competence, and relatedness drive intrinsic motivation. Our intervention enhanced autonomy (patients experienced fewer disruptive turns) and competence (real-time feedback empowered self-care awareness), which explains the significant increase in satisfaction in the study group. This aligns with SDT’s emphasis on psychological needs fulfillment in healthcare settings. The Effort-Recovery Model suggests that uninterrupted recovery periods prevent cumulative fatigue. By reducing repositioning frequency, our study indicated that nurses gained cognitive/physical recovery time during extended intervals. Freed resources were reallocated to high-value tasks (e.g., psychological support), mitigating “effort accumulation”—a key predictor of burnout. Based on the above theoretical framework, our study demonstrated that the novel technique of skin interface pressure and temperature monitoring in preventing pressure injuries significantly increased patients’ satisfaction and reduced nurses’ workload.

### 4.2 The use of pressure at the pressure interface, combined with skin temperature monitoring to guide the position management program monitors reduced the incidence of pressure injuries in paraplegic patients

Studies show that more than 20% of spinal cord injury patients develop pressure injuries due to motor and sensory dysfunction, limited physical activity, or prolonged periods of being bedridden or wheelchair-bound (14). Pressure injuries have a serious impact on the quality of life of people with spinal cord injuries, placing a heavy care and financial burden on their families and society; for example, the daily cost of treating pressure injuries ranges from 1.71 to 470.49€ per adult patient across different settings. In addition, the cost of treating severe pressure injuries can be even higher, and the prevention is more cost-effective than treatment (15, 16). Studies have shown that the risk factors of pressure injury persist after patients return to their families (17). Therefore, it is essential to know how to prevent postoperative pressure injury recurrence (18). Among the measures to prevent pressure injuries, interventions that reduce the intensity or duration of pressure are crucial for sedentary or bedridden patients (19). Inadequate postural changes are a key factor in the recurrence of pressure injuries. The comprehensive monitoring of patients with pressure injuries and the development of individualized protective measures are needed to reduce the recurrence rate of pressure injuries (20). Based on this study, the frequency and interval of position change for paraplegic patients were developed according to the interface pressure on the skin surface and skin temperature at the pressure interface. Dynamic postural adjustments can relieve pressure on the sacrococcygeal area promptly, preventing the pressure from exceeding the limit of capillary tolerance. This may facilitate reduced vascular occlusion, avoid abnormal changes in perfusion status, and ensure tissues receive sufficient oxygen and nutrient supply (21).

Our study showed that under the monitoring of the application of a position management program guided by the skin interface pressure combined with skin temperature monitoring, the incidence of pressure injuries at 3 months after the end of the trial was reduced from 6.7 to 2.3% (*p* = 0.616). More importantly, the incidence of PIs decreased from 13.3 to 4.5% (*p* = 0.266) and from 20.0 to 4.5% (*p* = 0.027) at 6 months and 12 months after the end of the trial. This suggests that combining skin interface pressure monitoring with skin temperature monitoring and the positional management program may contribute to a significant reduction in the incidence of pressure injuries in paraplegic patients in the long term.

In addition, Spearman’s correlation analysis revealed that the sacrococcygeal pressure values of paraplegic patients were dramatically positively correlated with the occurrence of pressure injuries. In contrast, the skin temperature values were negatively correlated with the occurrence of pressure injuries (all *p* < 0.05). This finding confirms that both sacrococcygeal pressure and skin temperature values can reflect pressure injuries in paraplegic patients at an early stage. These results suggest that this interventional strategy can serve as an early warning reference indicator for managing the risk of clinical pressure injury. It is worth noting that the occurrence of pressure injuries is closely related not only to active and effective positional management, but also to the patient’s age, severity of disease, limb mobility, cleanliness of the bed unit, nutritional support, and moistness of the pressurized skin (22). Therefore, multiple factors need to be considered when preventing pressure injuries in paraplegic patients to achieve a reduction in the risk of pressure injuries.

### 4.3 Pressurized interface pressure combined with skin temperature protocols can improve patient satisfaction and reduce nursing workloads

The nursing effect of position management for paraplegic patients using skin interface pressure combined with skin temperature monitoring shows that personalized position management not only does not increase the incidence of pressure injuries, but also reduces the patient’s discomfort and pain, facilitates the patient’s recovery, and improves the patient’s and family’s satisfaction. It also reduces the nursing staff’s time spent on position management for the patient and the number of position changes for the patient, thus reducing the nursing staff’s workload. The patients in the control group used the conventional 2-h position change frequency (all the patients were set to 2-h position change frequency). While in the study group, under the guidance of skin interface pressure combined with skin temperature monitoring, the frequency of change within 2 h was reduced to 4 persons, 2.1–2.5 h had 14 persons, 2.6–3.0 h had 13 persons, and 3.1–3.5 h had 5 persons. This finding shows that our interventional program has reduced the workload of the nursing staff to a certain extent. Recently, numerous studies have demonstrated that the use of wearable sensors has elevated compliance with frequent turn protocols, thereby reducing pressure injuries and facilitating a decrease in the organizational costs and nursing workloads (23). In this study, the novel monitoring device continuously monitors the temperature of the skin surface. This is because increased skin moisture and localized temperature are early warning signs of PIs development (24, 25). By monitoring this indicator, healthcare professionals can identify potential pressure sore risk areas in advance and take timely measures.

### 4.4 A robust monitoring protocol contained within the skin interface pressure combined with skin temperature is more in line with the new concepts advocated for high-risk pressure injury care

The 2019 edition of the PI guidelines demonstrated that the frequency of position changes should be determined according to the tolerance of the skin tissues for people at high risk of pressure injuries (26). It also pointed out that personalized schedules should be implemented for the position changes in patients. It was reported that skin interface pressure and skin temperature are highly significant predictors of reactive congestion indicators (*p* < 0.01). The skin interface pressure and skin temperature are two important indicators in the assessment of the tolerance of skin tissues (27). The guidelines also mention that the use of bedside pressure mapping as a visual cue to guide positional changes should be considered. Also, it is recommended to use the novel skin temperature detection devices to aid in skin assessment (28). This study focuses on paraplegic patients, a high-risk group for pressure injuries, and carries out the monitoring of skin pressure and skin temperature at the pressure interface, to reduce the intensity and duration of pressure at the pressure site of the paraplegic patient’s body. Based on this evidence, our study may provide the physiological early warning and monitoring indexes, coupled with local skin assessment and observation, so as to identify early skin changes of pressure injuries as early as possible. This strategy may prompt clinicians and nurses to take timely and effective interventions, which is in line with the new concepts of nursing care for high-risk pressure injuries advocated.

### 4.5 Strengths and limitations

This study possesses several key strengths, including its conduct in a real-world clinical setting across multiple departments (Department of Rehabilitation, Department of Orthopedics, Wound Center, and Department of Neurology) within two tertiary hospitals, enhancing the practical applicability of our findings. It features longitudinal follow-up at 6 months and 1-year post-trial, providing valuable data on long-term pressure injury outcomes. The research employed multiple outcome measures, assessing not only pressure injury incidence but also patient satisfaction and nursing workload, offering a comprehensive view of the intervention’s impact. It generated objective correlation data through Spearman’s analysis, demonstrating statistically significant relationships between sacrococcygeal pressure/temperature and pressure injury occurrence, thereby supporting the intervention’s physiological rationale. Critically, the study achieved statistically significant long-term results, showing a substantial reduction in pressure injury incidence at 12 months (4.5% vs. 20%). Furthermore, it documented significant benefits beyond injury reduction, including improved patient satisfaction and reduced nursing workload in the intervention group. Nevertheless, some limitations should be acknowledged when interpreting the findings of our study. The sample size of this study is small (n = 89), which may risk selection bias and restricts the generalizability of findings. This study was conducted in two Grade A tertiary hospitals, which may differ from those in primary-level hospitals or other regions, leading to limitations in the generalizability (applicability to broader populations) of research findings. Therefore, these factors may constrain the interpretability and relevance of the findings.

### 4.6 Implications for research and practice

Based on the main findings from our study, several implications for research and practice should be noted. First, our study may promote the clinical application of personalized positioning management. Personalized positioning protocols based on pressure and skin temperature monitoring can significantly reduce the incidence of long-term pressure injuries. This approach also minimizes unnecessary repositioning maneuvers (e.g., safely extending intervals to 3.5 h for selected patients), suggesting that the conventional rigid 2-h turning schedule can be replaced. Instead, protocols should be individualized according to patients’ specific pressure and skin temperature thresholds, thereby enhancing nursing precision. Second, we have established an early warning system for pressure injuries. Given that sacral pressure and skin temperature serve as valid predictive indicators, these parameters should be integrated into routine monitoring for paraplegic patients. Dynamic data tracking enables timely adjustment of care strategies (e.g., preemptive intervention for patients exhibiting high pressure or low skin temperature), facilitating early detection and timely prevention of pressure injuries.

## 5 Conclusion

This study demonstrates that personalized positioning management guided by real-time sacrococcygeal interface pressure and skin temperature monitoring significantly reduces long-term pressure injury incidence in paraplegic patients. Crucially, Spearman analysis confirmed sacral pressure (positively correlated) and skin temperature (negatively correlated) as statistically significant early-warning indicators, enabling proactive interventions. Our findings support replacing rigid 2-h turning schedules with individualized, physiology-driven protocols. Despite limitations, the evidence advocates integrating pressure–temperature monitoring into routine care for high-risk patients. This strategy aligns with international PI prevention guidelines by enabling early detection, resource-efficient interventions, and precision nursing.

## Data Availability

The datasets presented in this article are not readily available because we included the clinical data from the patients. Requests to access the datasets should be directed to zhouhuifen2562@163.com.
